# The Effect of Virtual Reality on Reducing Pain and Anxiety in Individuals Undergoing Arterial Blood Puncture

**DOI:** 10.7759/cureus.89035

**Published:** 2025-07-30

**Authors:** Fahad Somaa, Sultan Ahmed Balhmer, Mohammed Ahmed Alamri, Tareq Rabei Mominah, Fahd Mahmood Alhawsawi, Faisal Yahya Majhali, Azka Khan

**Affiliations:** 1 Occupational Therapy Department, Faculty of Medical Rehabilitation Sciences, King AbdulAziz University, Jeddah, SAU; 2 Occupational Therapy Department, Faculty of Medical Rehabilitation Sciences, King Abdulaziz University, Jeddah, SAU; 3 Respiratory Therapy Department, Faculty of Medical Rehabilitation Sciences, King Abdulaziz University, Jeddah, SAU; 4 Faculty of Rehabilitation and Allied Health Sciences, Riphah International University, Islamabad, PAK

**Keywords:** anxiety, arterial blood puncture, pain, virtual reality, vr distraction therapy

## Abstract

Background and objective

Arterial blood puncture (ABP) is a common, painful procedure frequently associated with anxiety. It is well-known that traditional methods of pain management do not provide relief in all cases, making it imperative to explore alternative methods like virtual reality (VR)-aided distraction. This study was designed to evaluate the influence of VR on pain and anxiety levels during ABP procedures.

Methods

A total of 79 adults were recruited to undergo ABP and were assessed using the State-Trait Anxiety Inventory (STAI) for anxiety and the Numerical Pain Rating Scale (NPRS) for pain. The VR intervention was performed before the second ABP. The data were analyzed with the help of SPSS Statistics, version 23 (IBM Corp., Armonk, NY), using paired sample t-tests.

Results

It was found that the VR intervention significantly decreased levels of pain (p<0.0005), with the average pain intensity improving from 4.21 ± 2.14 before intervention at baseline to 3.23 ± 1.88 after intervention. In contrast, there was no significant difference in anxiety levels before VR intervention versus after (p = 0.858).

Conclusions

VR-aided distraction has emerged as a non‐pharmacological method to reduce pain during ABP procedures. Further studies are required to determine its effect on anxiety and to evaluate its use in the broader clinical environment.

## Introduction

An arterial blood gas (ABG) test is a common and accurate method for checking blood gases and acid-base levels. It is often ordered in the ICU for monitoring blood gases and acid-base levels, making it one of the most requested tests. However, it can be expensive and may lead to various issues for the patient, like pain, infection risk, excessive blood loss, and rare but serious complications [1]. ABGs are seen as the most painful lab test and are typically done without using any pain relief [2]. Even though nursing and medical organizations suggest using local analgesia, healthcare providers are still reluctant. They worry that the injection of the anesthetic might be as painful as the arterial puncture, that applying the anesthetic might make it difficult to collect the sample, that administering the anesthetic takes too much time, or that the pain is comparable to that of a regular venipuncture [3,4].

In the past, various numbing medicines were used to lessen pain and make patients and doctors more comfortable. The goal was also to reduce the chances of complications and the risk of needle pricks. These numbing medicines were applied to the skin (like amethocaine) or injected (like lidocaine). While many skin-numbing creams take too long to work in the emergency room, lidocaine is seen as very helpful [5,6]. Doctors often avoid using it because they think it causes more pain when injected, causes delays in performing the procedure, and may change the appearance of the puncture site [5]. In the emergency department, an ideal anesthetic should work well, be non-invasive, and take effect immediately. WHO also advocates for adopting practical alternatives to proficiently manage pain in outpatient settings [7].

Pain management involves both pharmacological and non-pharmacological approaches [8]. Non-pharmacological methods, particularly beneficial for mildly invasive procedures, serve as valuable alternatives in alleviating pain [9]. Distraction, a non-pharmacological approach, directs attention away from pain toward a different stimulus. Employed to boost pain tolerance and reduce pain sensitivity, this method can be valuable in patient care [10]. Recently, virtual reality (VR) has been used in clinical settings to divert attention [11,12]. VR involves the development of a computer-generated, lifelike environment that delivers immersive and vivid experiences [13]. A study has shown that VR greatly reduced the procedural pain and anxiety of subjects aged 10-21 years during blood draws. Whether used alone or in combination with standard care, VR is effective in reducing pain and anxiety [14]. The study aims to explore the effects of VR on the pain and anxiety levels of individuals undergoing arterial blood puncture (ABP). The study also aims to contribute to improving patient well-being and healthcare experiences by investigating the potential of mitigating pain and anxiety in this context through the use of VR.

Although previous studies have demonstrated the effectiveness of VR in reducing pain during procedures such as venipuncture and those involving pediatric populations, there is a distinct lack of research specifically examining its application in ABP, a procedure known to be considerably more painful. This study directly addresses that gap by evaluating VR’s impact specifically in the context of ABP, a procedure that is both commonly used and more invasive than routine blood draws.

## Materials and methods

Study design

This was a quasi-experimental pre-post study. This study design was selected due to the ethical and logistical considerations of exposing participants to repeated ABPs purely for control purposes. To minimize potential biases from the lack of a control group, all procedures were standardized; the same respiratory therapist performed both ABPs, the time intervals were consistent, and validated tools were used to assess outcomes. The ethical approval for this study was obtained from the Ethics Research Committee at King Abdulaziz University with reference number FMRS-EC2024-029.

Participants

Participants aged 18-26 years were recruited for the study to ensure homogeneity in pain and anxiety perception, as these variables can vary widely across age groups. The participants were scheduled for ABP procedures at King Abdulaziz University Hospital. It was ensured that all participants had understood and followed instructions and expressed their willingness to use VR technology during the procedure. Participants who refused to participate or were not able to give informed consent were excluded. There were a total of 79 participants in the study. A convenience sampling technique was employed, recruiting participants from individuals scheduled for routine ABG procedures in the hospital during the study period.

Survey instrument

The State-Trait Anxiety Inventory (STAI) tool was used to measure the anxiety levels of the participants. Permission to use the STAI in the present study was obtained. The tool comprises 20 questions, and each question is rated on a scale of 1 (not at all) to 4 (very much so). Some items on the questionnaire are reverse-scored, meaning that higher responses indicate lower anxiety. These items are reversed before calculating the total score. Scores can range from 20 to 80, with higher values correlating with greater social levels and lower scores suggesting a mild degree of anxiety. The stability of the STAI scales was examined among high school and college students, both male and female, across various test-retest intervals, spanning from one hour to 104 days. The reliability coefficients demonstrated a reduction in magnitude as the intervals increased. The coefficients for the Trait-anxiety scale ranged from .65 to .86, whereas the State-anxiety scale had coefficients between .16 and .62. The lower stability observed in the State-anxiety scale was anticipated, as its items are believed to reflect the impact of short-term situational factors during testing [15].

The Numerical Pain Rating Scale (NPRS) was used to measure pain. This is a simple and typical means of measuring pain intensity, where the subject selects a number ranging from 0 to 10 to correspond to the amount of pain felt. The scale is divided into ranges of mild (1-3), moderate (4-5), severe (6-7), very severe (8-9), to the worst possible pain (10). It is a structured and quantifiable measure of pain intensity, reducing the complexity of pain interpretation as well as comparison of different people and environments [16].

Procedure

The Ethical Committee at King Abdulaziz University authorized the necessary ethical approval for this investigation. The survey was voluntary, while researchers established strict procedures to protect the anonymity of participant responses. Before starting the survey, all participants received an informed consent form, which they needed to review and sign. Each survey participant received a 23 G1 ABG syringe produced by BD Preset (BD, Franklin Lakes, NJ). All arterial punctures were performed by one respiratory therapist. Modified Allen tests were performed for all participants to assess blood flow and the quality of the radial pulse. Pain and anxiety were assessed after the arterial punctures by using NPRS and STAI for the first puncture attempt, with no distraction method applied. Then, participants rested for five minutes.

Participants wore the VR headset from Oculus and immersed themselves in the chosen experience, which was accompanied by a video of the Maldives paradise and tropical beach relaxation to complement the visual experience and enhance distraction and relaxation. After wearing the VR headset, a three to five-minute wait period ensued, and the second ABG was drawn. All second ABG samples were collected from the contralateral radial artery to minimize any localized desensitization, inflammation, or tissue sensitization effects from the initial puncture. Studies by Fowler et al. [17] and Chad et al. [18] suggest that even short VR sessions lasting three to five minutes can effectively reduce perceived pain during medical procedures. Pain and anxiety were assessed again using NPRS and STAI. An exploratory analysis comparing pain levels between male and female participants was also conducted to identify potential gender-based differences in response to VR intervention.

Data analysis

The data were analyzed using SPSS Statistics, version 23 (IBM Corp., Armonk, NY), beginning with an evaluation of its normality to select the appropriate statistical tests. Cohen’s d was calculated for the reduction in pain levels following the VR intervention. The effect size was found to be 0.49, indicating a moderate practical significance of the VR intervention in reducing pain. Descriptive statistics were applied, and a paired sample t-test was performed to compare the pre- and post-intervention mean values for anxiety and pain. An independent t-test was conducted to compare pain scores between genders post-VR intervention. The test was appropriate, given the normal distribution confirmed via the Shapiro-Wilk test. A p-value >0.05 was considered statistically significant.

## Results

Descriptive statistics

**Figure 1:**
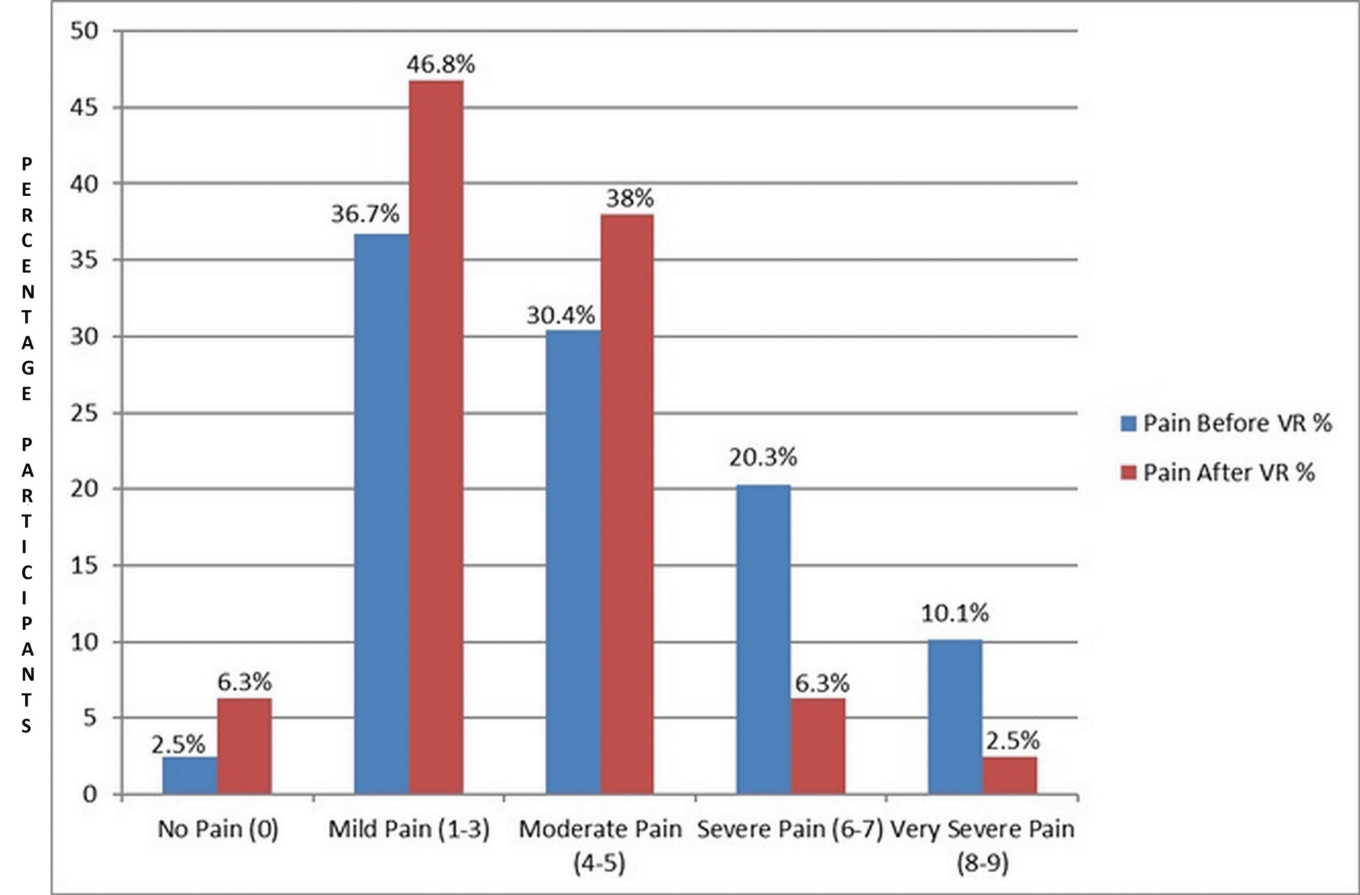
Pain levels before and after VR intervention (N = 79) VR: virtual reality

Of the total sample size of 79 individuals, 53 (67.1%) identified as male and 26 (32.9%) as female. The mean anxiety levels before and after VR intervention were 46.6 ±6.6 and 46.8 ±6.2, respectively. These findings suggest minimal change in anxiety levels following the VR session. In contrast, participants reported an average pain intensity of 4.2 ±2.1 before VR, which notably decreased to 3.2 ±1.8 after VR. This reduction in pain intensity post-VR underscores the potential therapeutic benefit of VR in pain management. The analysis of pain levels before and after VR intervention revealed notable shifts in participants' reported pain intensity. Before engaging in VR, the majority of participants experienced some degree of pain, with mild pain being the most prevalent at 29 (36.7%). However, after undergoing VR intervention, mild pain remained the most common, with a slightly higher frequency at 37 (46.8%), while moderate pain decreased to 30 (38.0%) (Figure 1).

**Figure 2:**
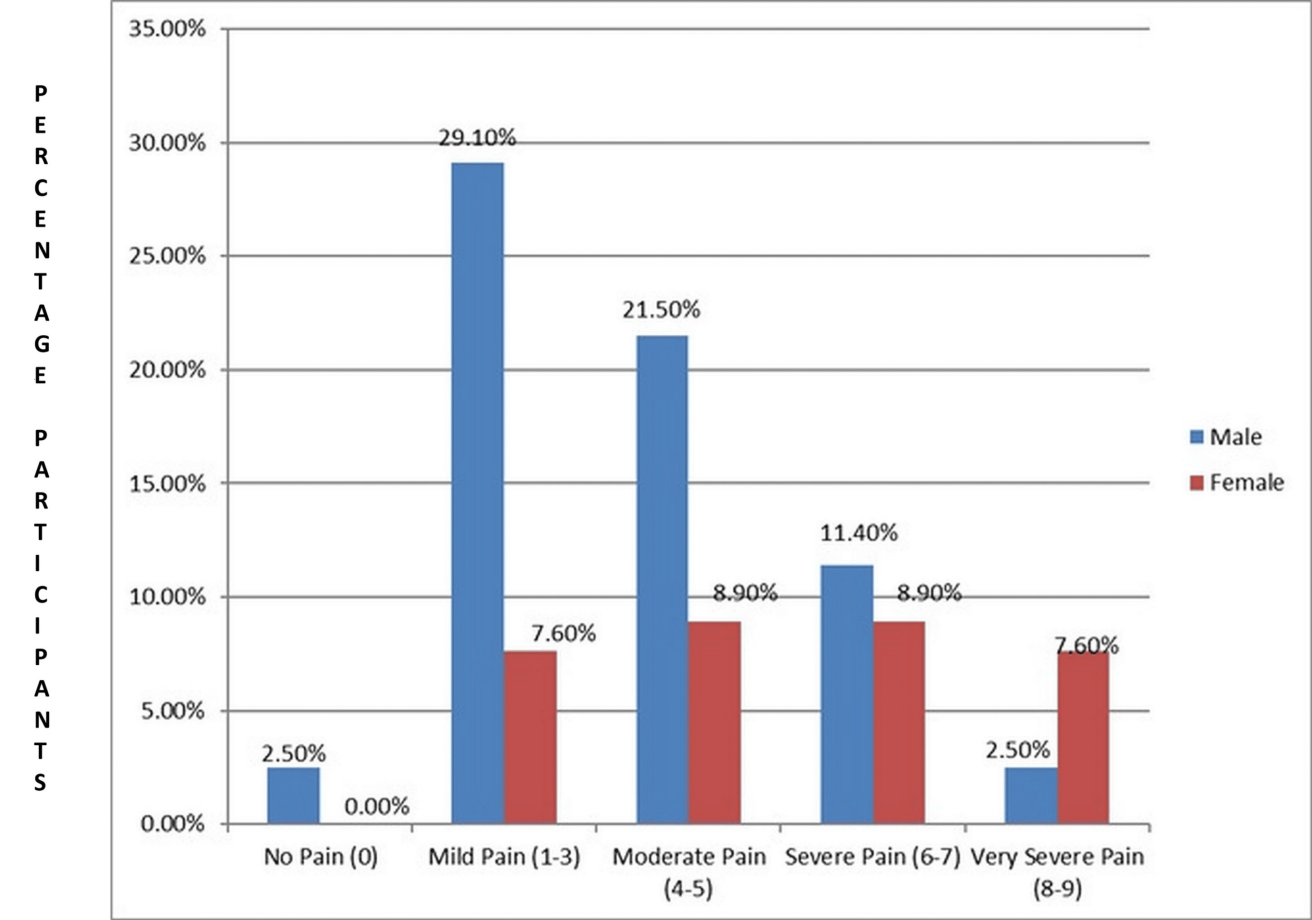
Pain before VR intervention in males and females (N = 79) VR: virtual reality

The analysis of pain levels reported by participants before engaging in VR intervention unveils distinct patterns between male and female respondents. Among male participants, a notable 23 (29.10%) reported experiencing mild pain (rated between 1-3 on the pain scale). In contrast, female participants exhibited lower levels across all pain categories, with only six (7.60%) reporting mild pain. These findings suggest a potential gender discrepancy in pain perception, with females generally reporting lower levels of pain before VR engagement compared to males (Figure 2).

**Figure 3:**
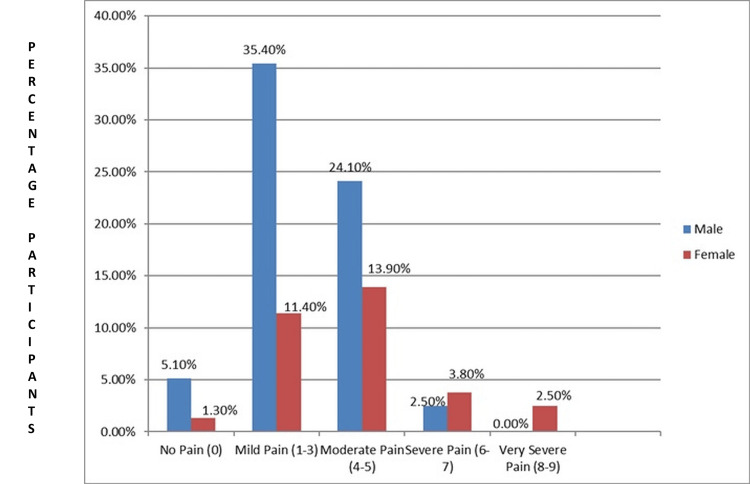
Pain after VR intervention in males and females (N = 79) VR: virtual reality

The examination of pain levels reported by participants after the VR intervention reveals notable differences in the experiences of male and female respondents. Among male participants, 28 (35.4%) reported experiencing mild pain (rated between 1-3 on the pain scale) after the VR session. Conversely, female participants exhibited lower levels across these categories, with nine (11.4%) reporting mild pain. These findings suggest that while both genders experienced varying degrees of discomfort after VR engagement, females generally reported lower levels of pain compared to males, albeit with a slightly higher prevalence of very severe pain among females (Figure 3).

The Shapiro-Wilk test indicated that the data for both anxiety and pain scores were normally distributed (p>0.05). Based on this, a paired-sample t-test was conducted to compare the means of the two related groups and identify any differences. The results showed no statistically significant change in anxiety levels before and after VR intervention, t(78) = -0.179, p = 0.858. However, the paired-sample t-test demonstrated a significant reduction in pain levels after VR engagement, t(78) = 4.313, p<0.0005. Pain decreased from 4.21 ±2.14 before VR intervention to 3.23 ±1.88 after, indicating a mean reduction in pain of 0.98 ±2.03 (Table 1).

**Table 1:** Paired sample t-test results for anxiety and pain levels (N = 79) df: degree of freedom; VR: virtual reality

Variable	Mean before VR	Mean after VR	Mean difference	Standard deviation	t-value	df	P-value
Anxiety	46.60	46.80	-0.20	6.89	-0.179	78	0.858
Pain Intensity	4.21	3.23	0.98	2.03	4.313	78	<0.0005

## Discussion

In this study, significant changes in participants' reported pain intensity were observed when comparing pain levels before and after the VR intervention. Before VR use, the majority of participants experienced some level of pain, with mild pain being the most prevalent (36.7%; n = 29), followed closely by moderate pain (30.4%; n = 24). A smaller percentage of participants (10.1%, n = 8) reported very severe pain, while a notable proportion (20.3%, n = 16) reported severe pain. However, there was a noticeable decrease in reported pain levels following VR intervention. The frequency of moderate pain decreased to 38.0% (n = 30), while mild pain remained the most common, albeit with a slightly higher frequency of 46.8% (n = 37). Importantly, after VR use, the proportion of participants reporting no discomfort increased to 6.3% (n = 5), indicating a beneficial effect of the intervention. Following VR, the percentage of participants reporting severe and very severe pain decreased to 2.5% (n = 2) and 6.3% (n = 5), respectively.

Additionally, a 2019 study on chronic pain showed the effect of VR on alleviating anxiety [17]. Another study by Chad et al. reported pain reduction during the blood draw experience [18]. Discrepancies in findings across studies may be due to variations in procedural invasiveness, patient demographics, and the nature of the VR content. The VR interventions involving dynamic or interactive environments have shown greater impact, whereas passive viewing may be less effective during high-stress procedures. In contrast, Malloy and Milling observed that while needle-related studies were less reliable, VR was a successful diversion for lowering pain and discomfort [19]. Nilsson et al. found no statistically significant changes between the experimental (VR) group and the control group in a study on pain connected to needles [20]. Likewise, a study by Glenno et al. found that the use of VR during medical procedures did not negatively impact patients' experiences or increase their pain levels. Furthermore, patients expressed satisfaction with using the VR eyewear. According to a nurse involved in the study, "Patients using the goggles appear to be less anxious about their surroundings, providing them with a brief respite [21]. Wint et al. concluded that VR was a workable, non-invasive intervention in patients [22].

In the present study, anxiety levels were 46.6 ±6.6 and 46.8 ±6.2, on average, before and after the VR intervention. These results imply that anxiety levels changed very little after the VR session. The minimal change in anxiety scores suggests that a short-duration VR exposure may not be sufficient to elicit a measurable reduction in state anxiety, which is likely influenced by a range of situational and psychological factors beyond sensory distraction. On the other hand, the average pain intensity indicated by the participants before VR was 4.2 ±2.1, and it significantly dropped to 3.2 ±1.8 following VR. This decrease in pain intensity following VR emphasizes VR's potential therapeutic value in pain treatment. Similarly, in an outpatient study, Brown et al. found that patients who experienced VR immersion scored less anxiously than the controls [23]. According to a review study on venous catheter placement by Malloy and Milling, using VR technology could help lessen discomfort and anxiety during the process [19]. A review found the effectiveness of anxiolysis to be ambiguous, noting that 50% (9 out of 18) of the studies reported a significant anxiolytic effect. A recent systematic analysis indicated that patients undergoing VR therapy for anxiety disorders experienced significantly lower anxiety levels compared to control groups [24]. Furthermore, evidence suggests that VR therapy not only enhances mood and fosters positive emotions but also helps reduce anxiety in patients before procedures. It is also hypothesized that these psychological changes may lead to anxiolytic effects that resemble the therapy's analgesic properties [25,26].

The present study also demonstrated that the experiences of male and female participants differ substantially in their reported pain levels after participating in VR activities. Results showed that males reported mild discomfort on average (receiving scores 1-3 in the pain scale), amounting to 35.4% of all participants. Additionally, 24.1% of males experienced moderate pain (ranking at 4-5 level). In contrast, the percentage of female participants who reported light pain was 11.4%, while the proportion of individuals who reported moderate pain was 13.9%. It is interesting to note that just a small percentage of respondents (2.5% of men and 3.8% of women) reported having extreme pain (rated 6-7). However, females were represented more in the very severe pain group (rated between 8 and 9), with 2.5% reporting such extreme discomfort compared to 0.0% of males. Furthermore, 5.1% of males and 1.3% of females reported no pain after the VR session.

These findings indicate that while both genders experienced differing levels of discomfort following VR intervention, females, while generally having fewer cases of these pain levels, displayed a slightly higher incidence of very severe pain. However, it is worth noting that there are no studies in the current literature on VR demonstrating that pain levels are consistently greater in either males or females.

## Conclusions

Our findings highlight the potential of VR intervention to alleviate pain during ABP procedures. We observed a significant reduction in reported pain intensity following VR engagement, with participants experiencing a notable decrease from moderate to mild pain levels on average. Although anxiety levels showed minimal change before and after VR, the impact on pain management underscores the therapeutic potential of immersive VR experiences in clinical settings. Furthermore, the study demonstrates that VR users of both genders reported different extents of pain before VR participation, which changed significantly after their VR experience. Before VR, males indicated more mild, moderate, and severe pain than females did, where females generally had fewer cases of these pain levels, yet displayed a slightly higher incidence of very severe pain. VR exposure led to decreased pain intensity for men and women, yet males experienced a bigger decrease in their experience of pain. Female pain levels decreased across various categories, although the proportion of cases with very severe pain remained higher among them compared to their male counterparts. The relatively small sample size of the study may limit the generalizability of the findings. Future studies should investigate the long-term benefits of VR-aided distraction, preferably by adopting a longitudinal design.
